# Overexpression of AmpC Promotes Bacteriophage Lysis of Ampicillin-Resistant *Escherichia coli*

**DOI:** 10.3389/fmicb.2019.02973

**Published:** 2020-01-08

**Authors:** Shuang Wang, Bo Yin, Ling Yu, Mei Dang, Zhimin Guo, Guangmou Yan, Dongliang Hu, Jingmin Gu, Chongtao Du, Xin Feng, Wenyu Han, Yuren Yuan Adam, Changjiang Sun, Janine T. Bossé, Liancheng Lei

**Affiliations:** ^1^College of Veterinary Medicine, Jilin University, Changchun, China; ^2^Center for Bioengineering and Biomedical Sciences, National University of Singapore (Suzhou) Research Institute, Jiangsu, China; ^3^Department of Respiratory Medicine, The Second Hospital of Jilin University, Changchun, China; ^4^Department of Zoonoses, Kitasato University School of Veterinary Medicine, Towada, Japan; ^5^Department of Infectious Disease, Imperial College London, London, United Kingdom; ^6^College of Animal Science, Yangtze University, Jingzhou, China

**Keywords:** bacteriophage, antibiotic resistance, ampicillin, *E. coli*, AmpC

## Abstract

Infections caused by antibiotic-resistant *Escherichia coli* are a threat to human and animal health globally. Phage therapy has made great progress for the treatment of drug-resistant infections, but it is still unclear whether *E. coli* resistance to antibiotics could change the lysis ability of phages. In this study, we demonstrate that over expression of AmpC, an important β-lactamase for ampicillin resistance, promotes lysis of *E. coli* by phage utilizing OmpA as a receptor. *E. coli* strains expressing more AmpC showed higher levels of OmpA, an *E. coli* outer membrane protein known to serve as a receptor for T-even phages, which resulted in increased adsorption and lysis by the phage tested in this study. These data demonstrate that increased ampicillin resistance can increase the sensitivity of *E. coli* to some lytic phage, which provides evidence for the feasibility of synergistic application of phage and antibiotics.

## Introduction

*Escherichia coli* is an important foodborne opportunistic pathogen, and acquired antibiotic resistance is increasing the global threat. The World Health Organization has listed 12 drug-resistant pathogens of greatest concern for human health, the most important of which are *Acinetobacter baumannii*, *Pseudomonas aeruginosa*, and *Enterobacteriaceae* (Tacconelli et al., 2017). Antibiotic resistance accounts for 700,000 deaths every year, and is estimated to reach 10 million by 2050 if there is no reduction in antimicrobial resistance or development of new antibiotics (Willyard, 2017). There is now an increased focus on alternative biotherapies (Thiel, 2004; Kelly et al., 2016).

Bacteriophages are regarded as potential biotherapeutic agents in the fight against resistant bacteria (Matsuzaki et al., 2014). Phage therapy has many advantages over the current use of antibiotics, such as reducing the incidence of recurrence after cessation of treatment, protecting the host from excessive intestinal growth of other pathogens, and diminished resistance to metronidazole or vancomycin (Ramesh et al., 1999). Furthermore, phage-lysed bacteria release less endotoxin than antibiotic-treated bacteria (Dufour et al., 2017). Phage replication at the site of infection enhances efficacy; a single intraperitoneal injection of phage ENB6 rescued 100% of mice with vancomycin-resistant enterococci bacteremia (Biswas et al., 2002). Since long-term use of phage is not known to produce any side effects, it can be used for the treatment of chronic infectious diseases (Drilling et al., 2017). Selection for phage-resistant isolates may occur, but this is limited to the target bacterial species of the phage used for treatment. In contrast, prolonged antibiotic treatment may select for increased resistance amongst all exposed bacteria (pathogens and commensals) not only for the antimicrobial agent used, but also any other agents for which the resistance genes are co-selected (Murray et al., 2018). Whether bacteriophages can effectively lyse antibiotic-resistant bacteria remains unclear.

β-Lactam drugs play an important role in fighting infections caused by Gram-negative bacteria. However, the spread of β-lactamases (in particular those with broad spectrum of activity) in pathogenic bacteria has become a serious problem, increasingly leading to treatment failures (Bai et al., 2017). Chromosomally encoded AmpC is present in many *Enterobacteriaceae* species and confers resistance to multiple β-lactam antibiotics (Fisher et al., 2005; Jacoby, 2009). In some species, the release of cell wall degradation products upon exposure to β-lactams contributes to a complex regulatory pathway involving numerous proteins, culminating in high level expression of *ampC* following displacement of the repressor, AmpR (Jacoby, 2009; Pérez-Gallego et al., 2016). Inducible expression of chromosomal *ampC* does not occur in *E. coli*, which lacks AmpR (Jacoby, 2009). However, hyperproduction of AmpC in *E. coli* has been associated with mutations in the promoter and/or attenuator regions upstream of *ampC*, as well as with the presence of extra copies of the gene, either in the chromosome or on acquired plasmids (Nelson and Elisha, 1999; Peter-Getzlaff et al., 2011).

Resistance to β-lactams is also affected by alterations in the bacterial outer membrane which affect uptake of antibiotics, in particular, changes in expression profiles of the major porins. Some β-lactam resistant clinical isolates of *E. coli* have been found to lack OmpF in addition to hyperproducing AmpC (Martinez-Martinez et al., 2000). Recently, Choi and Lee (2019) constructed mutants of all porins in *E. coli* and confirmed that OmpF is the main porin associated with uptake of antibiotics, especially β-lactams, with OmpC contributing to a lesser extent. In contrast, loss of OmpA was associated with increased sensitivity to antibiotics (Choi and Lee, 2019). It is not yet known if antibiotic-induced changes in outer membrane profiles of Gram-negative bacteria affect their sensitivity to phage.

In previous studies, we have identified and characterized two related phage, vB_EcoM-ep3 and vB_EcoM_ECOO78, both *Myoviridae* capable of infecting a number of multiple-drug resistant *E. coli* clinical isolates (Lv et al., 2015; Guo et al., 2017). In this study, we show that hyper-production of AmpC is directly related to decreased OmpF, and increased OmpA, expression in *E. coli* and that these changes are associated with increased adsorption and lysis by these phages. Our findings provide a viable basis for the use of phage therapy for bacterial infections, in particular for treatment of β-lactam resistant bacterial infections.

## Materials and Methods

### Bacterial Strains, Bacteriophages, and Growth Condition

*Escherichia coli* strains and their sources are listed in Table 1. Phage vB_EcoM-ep3 and vB_EcoM_ECOO78 were isolated previously in our laboratory (Lv et al., 2015; Guo et al., 2017); all other phages were isolated from hospital or domestic sewage, or water from rivers and lakes around Changchun, either as part of this or a previous study in our laboratory. Except for phage vB_EcoM-ep3 and vB_EcoM_ECOO78, the remaining phages were not characterized in depth. Phages and their host spectra are listed in Supplementary Table S1. Unless otherwise stated, *E. coli* was grown in 3 mL LB broth (1% [wt/vol] tryptone, 0.5% [wt/vol] yeast extract, 1% [wt/vol] NaCl) at 37°C. In order to proliferate phage, 100 μL of fresh logarithmic host bacteria were inoculated into LB broth, and a single phage plaque was scraped from a double-layer plate, prepared as previously described (Barrow et al., 1998), using a sterile loop and inoculated into the above medium. The culture was incubated at 37°C, with shaking (180 rpm), until the host bacteria were completely lysed to make the medium clear (about 2–6 h but varies for different phages).

**TABLE 1:** Information of *E. coli* strains and number of corresponding phages.

		**Number of**			**Number of**
	**MIC**	**corresponding**		**MIC**	**corresponding**
***E. coli* strains**	**(ampicillin mg/L)**	**phages**	***E. coli* strains**	**(ampicillin mg/L)**	**phages**
*E. coli* HXM^ a^	>800	1	*E. coli* TSQ^a^	>800	1
*E. coli* LSZ^ a^	>800	1	*E. coli* ZDY^a^	>800	1
*E. coli* CZ ^a^	>800	3	*E. coli* O78-3^b^	>800	4
*E. coli* ZLH^ a^	>800	3	*E. coli* O78-6^b^	>800	5
*E. coli* WZ^ a^	>800	4	*E. coli* CC11^b^	>800	6
*E. coli* SGF^ a^	>800	1	*E. coli* GYP^a^	>800	2
*E. coli* O78-5^ b^	>800	2	*E. coli* S-F23^c^	>800	4
*E. coli* CVCC1418AmpR	>800	4	*E. coli* ATCC25922^d^	25	3
*E. coli* CVCC1418^ e^	25	3	*E. coli* K12 MG1655^f^	25	2
E coli ZDZ^ a^	25	3	*E. coli* MYL^ a^	25	2
*E. coli* LHY^ a^	25	1	*E. coli* ZZJ^a^	25	2
*E. coli* 84^ a^	25	1	*E. coli* BDY^a^	25	1
*E. coli* YFX^ a^	25	1	*E. coli* PJ^a^	25	4
*E. coli* TC^ a^	12.5	2	*E. coli* YXM^a^	12.5	1
*E. coli* MLD^ a^	12.5	2	*E. coli* ZX^a^	12.5	1
*E. coli* DQQ^a^	12.5	3	*E. coli* WHL^a^	<6.25	3
*E. coli* SWD^ a^	<6.25	2	*E. coli* BL21(DE3)^g^	<6.25	2

*^*a*^Isolated from patients at the First Hospital of Jilin University (Changchun, China); ^*b*^Isolated from chicken of Feng Xin chicken farm in Chuangchun; ^*c*^Isolated from swine lung from Changchun farm; ^*d*^Purchased from the ATCC; ^*e*^Purchased from the China veterinary culture collection center; ^*f*^Purchased from China biovector plasmid vector bacterial cellular protein antibody gene collection center. ^*g*^Purchased from Tiangen Biotech (Beijing), Co., Ltd.*

### Minimum Inhibitory Concentration (MIC) Assay

Minimum inhibitory concentration of ampicillin were determined by standard methods (Wiegand et al., 2008) for all *E. coli* strains used in this study. A single colony of each *E. coli* strain was inoculated into fresh LB medium and cultured to logarithmic growth phase; 2 μL of the bacterial culture were inoculated into 200 μL CAMHB medium containing ampicillin at final concentration of 800, 400, 200, 100, 50, 25, 12.5, or 6.25 mg/L and cultured at 35°C for 18 h, after which the bacterial growth was observed.

### Host Range Determination

The 81 phages shown in Table 1 were tested, using double-layer plate assay, for their ability to lyse the 29 clinical *E. coli* isolates (from human or animal sources), as well as strains obtained from culture collections, i.e., ATCC25922 (antibiotic sensitive serotype O6 reference strain), BL21(DE3) and K12 MG1655 (both antibiotic-sensitive rough strains, lacking O polysaccharide chains), and CVCC1418 (an antibiotic-sensitive O78-type *E. coli* strain). In addition, a spontaneous mutant of the latter strain, CVCC1418AmpR, with high resistance to ampicillin was selected following serial passage in LB broth containing increasing concentrations of ampicillin (from 1 to 1024 mg/L). This mutant strain was also tested for lysis with the panel of phages. For the double-layer agar plate test (Barrow et al., 1998), the phage dilution and the tested *E. coli* strain were added to the melted semi-solid medium (45°C) and immediately mixed, then poured onto LB agar plates and cultured at 37°C for 10 h. The formation of plaques was observed following over-night incubation of the plates at 37°C.

### Bacterial Lysis Assay

A bacterial lysis assay was performed following a published procedure (Ronayne et al., 2016) with some modifications. Briefly, logarithmic phase cultures of *E. coli* were diluted 1:100 in fresh LB and grown at 37°C to an optical density at 600 nm (OD_600_) of at least 0.55. Within each experiment, cultures were adjusted to an equal OD_600_ (0.5 to 1.2) and phage was added at a multiplicity of infection (MOI) of 10. Control cultures were left uninfected. Every 20 min, over a 2 h period, 200 μL aliquots were removed to measure the OD_600_ using BioPhotometer plus (Eppendorf). Phage lysis rate was calculated as [1-(the OD_600_ of phage treated group/the OD_600_ of control group)] × 100% after phage treatment for 80 min. Three biological replicates were performed, and the data is represented as mean with standard deviation.

### Adsorption Rate Assay

Phage absorption was assessed as described previously (Park et al., 2017), with some modifications: mid-exponential phase recipient *E. coli* strains were mixed with phage lysate in phage buffer (50 mM Tris-HCl pH 7.5, 100 mM NaCl, 10 mM CaCl_2_) at a MOI of 10 and incubated for 20 min at 37°C. Phage bound to bacterial cells were removed by microcentrifugation of 1 mL of the infected culture at 13,000 × *g* for 15 min at 4°C. The plaque-forming units (PFUs) of the supernatant containing the unbound phage was determined by the double-layer agar plate test. The phage absorption (% input) was calculated as [1 - (the number PFU of unbound phage/input PFU)] × 100%. Each absorption assay was repeated at least three times.

### Genetic Manipulations

Bacterial genomic DNA was extracted using the Bacterial DNA Kit (Omega, Norcross, GA, United States), and phage DNA extracted using Viral DNA Kit (Omega, Norcross, GA, United States). Plasmids were isolated using 96 Well Plasmid DNA Mini-Preps Kit (Sangon Biotec, China). Restriction endonucleases and T4 DNA ligase were purchased from Thermo Scientific and used according to manufacturers’ instructions. Premix Taq^TM^ (Ex Taq^TM^ Version 2.0) (TAKARA, # RR003Q, Japan) was used to amplify AmpC, OmpA, and Dpo41. Primers used are listed in Supplementary Table S2. Sequence analysis was performed by Genewiz^1^.

### pET-23a-*ampC* Construction for Over-Expression of AmpC and Complementation

The coding sequence of *ampC* was amplified from *E. coli* strain K12 MG1655 genomic DNA using primers AmpCF/AmpCR. Sequence was digested with *Bam*HI and *Eco*RI and inserted downstream of the T7 promoter in the plasmid pET-23a to produce pET-23a-*ampC*, and the resulting plasmid was transformed into *E. coli* DH5α by electroporation. Clones were selected on LB with kanamycin (34 mg/L) and insertions were confirmed by sequencing using primers AmpCF/AmpCR.

For overexpression of AmpC, the plasmid pET-23a-*ampC* was introduced into *E. coli* strain CVCC1418, where it was stably maintained. The bacterial culture was harvested by centrifugation and re-suspended in ice-cold lysis buffer (25 mM Tris pH 7.4, 25 mM KH_2_PO_4_, 500 mM NaCl, 10% glycerol and 1 mM dithiothreitol). Cell suspensions were disrupted using an EmulsiFlex-C3 high-pressure homogenizer (Avestin) four times and centrifuged at 13,000 × *g* for 1 h at 4°C to remove cell debris. The soluble cytoplasmic proteins in the resulting supernatant were identified by SDS-PAGE.

The same plasmid, pET-23a-*ampC*, was used to complement the *ampC* deletion mutant of *E. coli* strain K12 MG1655, described in the next section. The original plasmid pET-23a was used as a complementation control. Plasmids propagated in *E. coli* strain DH5α were transformed into *E. coli* strain K12 MG1655Δ*ampC.*

### Strain K12 MG1655Δ*ampC* Construction

Gene knockout was performed as described previously (Jiang et al., 2015). Following unsuccessful attempts to delete *ampC* in strain CVCC1418, CRISPR/Cas9 was used to knock out *ampC* in *E. coli* strain K12 MG1655. In this two-plasmid system, the cas9 gene and the sgRNA directing it to the targeted region were separated into pCas (Addgene: #62225) and pTargetT-Δ*ampC*. The latter plasmid was constructed by inserting the donor DNA used as the genome editing template into pTargetF (Addgene: #62226), followed by N20 sequence mutation via inverse PCR using the KOD-plus-neo polymerase (TOYOBO, Osaka) with primers pAmpC01/pAmpC02. The genome editing template was concatenated by a 550-bp sequence homologous to each side of the *ampC* gene through overlap PCR of the two fragments amplified from K12 MG1655 using primers pUAmpC1/pDAmpC1 to form the upstream, and pUAmpC2/pDAmpC2 to form the downstream, region. Strain K12 MG1655 competent cells harboring pCas were prepared and the plasmid pTargetT-Δ*ampC* was then introduced both by electroporation (2.5 kV), as described previously (Jiang et al., 2015). Cells were recovered at 30°C for 2 h before being spread onto LB agar containing kanamycin (34 μg/mL), spectinomycin (50 μg/mL), and arabinose (10 mM) and incubated 16 h at 30°C. Colonies harboring both pCas and pTargetT-Δ*ampC* were picked randomly and inoculated into 5 mL LB medium with the addition of kanamycin (34 μg/mL) and isopropyl β-D-thiogalactoside (IPTG) (0.8 mM). After 24 h cultivation, the culture was diluted and spread onto LB agar plates containing kanamycin (34 μg/mL) and IPTG (0.8 mM). The colonies were confirmed as cured by determining their sensitivity to spectinomycin (50 μg/mL). The colonies cured of the TargetT-Δ*ampC* were used for extraction of genomic DNA, and they were validated by PCR using primers pUAmpC1/pDAmpC2 and DNA sequencing. For the curing of pCas, an edited colony harboring pCas was inoculated into LB medium at 37°C non-selectively.

### Western Blot Analysis of AmpC Expression

Western blotting was performed as described previously (Ballesteros-Plaza et al., 2013) with some modifications: *E. coli* cells were sonicated. *E. coli* cell (1 × 10^8^ CFU) lysates were boiled in 5× loading buffer [0.25M Tris-HCL (pH 6.8); 10% (w/v) sodium dodecyl sulfate; 0.5% (w/v) bromophenol blue; 50% (v/v) glycerol; 5% (w/v) β-mercaptoethanol], and proteins were separated by SDS-PAGE and transferred to polyvinylidene difluoride membranes using eBlot Protein Transfer Device (Genscript), according to manufacturer’s instructions. Membranes were probed with anti-*E. coli* AmpC Ab (mouse polyclonal antiserum; for details of preparation, see Supplementary Data) and anti-GAPDH Ab (1:1000 in PBS, rabbit polyclonal, #E-AB-20059, from Elabscience) followed by horseradish peroxidase (HRP)-conjugated goat anti-mouse Antibody (1:5000 in PBS, #bsm-0294M-HRP, Bioss, China). Chemiluminescence was developed using an Immobilon Western chemiluminescent HRP substrate (Millipore) and documented using a Tanon 5200 milti (Biotanon). Densitometry was performed using ImageJ software (Ver 1.4.3.67) and was normalized to GapA (probed by anti-GAPDH Ab) (Wu et al., 2012).

### iTRAQ Proteomics

iTRAQ analysis was performed at Beijing Qinglian Bio Biotechnology, Co., Ltd. (BQB, Beijing, China). The protocol was as follows, *E. coli* strain CVCC1418 and CVCC1418AmpR were cultured to logarithmic phase. After centrifugation at 3000 × *g* for 5 min at room temperature, cells were washed three times with a sterile PBS solution, cells and collected after removing the PBS solution. Samples (wet weight is 400 μg) were resuspended in 700 μL of lysis buffer (7M urea, 2M thiourea, 0.1% CHAPS) and mixed by vortex. Sample was sonicated using an ultrasonic cell disruptor (0.2 s bursts every 2 s for 60 s, at an amplitude of 22%, Nanjing Atpio, Cat No: XO) and incubated at room temperature for 30 min, followed by centrifugation at 13,000 × *g* for 4 min at 4°C. The protein concentration of the supernatant was determined by Bradford Assay (Bradford, 1976). Enzymatic hydrolysis and labeling was performed using the iTRAQ kit (AB Sciex, PN: 4381664), according to manufacturer’s instructions. Primary separation of digested peptides and LC-MS/MS analysis (Thermo Q-Exactive mass spectrometer) were performed after that, using default parameters. Proteins with a difference of ≥1.2, or a difference of ≤0.833, were analyzed. Gene Ontology (GO) functional annotation (Dessimoz and Škunca, 2017), functional enrichment analysis, and localization analysis were performed on the identified proteins using the bioinformatics analysis tool DAVID (Huang et al., 2007). *P*-value calculation formula for the significant enrichment of the differential protein in a certain GO functional category is as follows:

(1) $$pValue = 1 –\sum_{k=0}^{n-1}\frac{\left(\begin{array}{c} n-k\\ N-M \end{array}\right)\,\left(\begin{array}{c} k\\ M \end{array}\right)}{\left(\begin{array}{c} n\\ N \end{array}\right)}$$

In the formula (1), N is the total number of proteins annotated by GO; M is the number of proteins belonging to a certain GO subclass; n is the number of protein sets subjected to GO enrichment analysis; and k is the number of proteins belonging to M in n.

### Phage Receptor Binding Protein (RBP) Identification

To identify the RBP of phage vB_EcoM-ep3, we used a previously published method (Simpson et al., 2016). Briefly, extracted phage genomic DNA was broken using ultrasound waves into random fragments of 150 to 2500 bp. Following end-filling using Quick Blunting^TM^ Kit (NEB, #E1201L) and phosphorylation using Alkaline Phosphatase (NEB, #M0290S), the purified random phage DNA fragments were ligated into *Eco*RV-digested pET-30a vector and transformed into BL21 (DE3) competent cells, with positive clones selected on LB plates containing kanamycin (34 μg/mL). Phage protein fragments expressed by the resulting positive clones capable of binding to *E. coli* O78-6 were recognized as encoding RBPs.

### Phage Blocking Experiment

For the phage blocking assay, 1 × 10^9^ PFU of phage vB_EcoM-ep3 were pretreated with 10 μL anti-Dpo41 serum or GST-OmpA (1 mg/mL) for overnight at 4°C, culture of *E. coli* strain O78-6 in logarithmic growth phase was added (MOI of 10). The calculation of the adsorption rate and the lyse rate were as described above.

### Co-immunoprecipitation (Co-IP)

The Co-IP experiments were performed using a Pierce^TM^ Classic Magnetic IP/Co-IP Kit (Thermo Fisher, #88804) following the manufacturer’s instructions. Briefly, His-Dpo41 and GST-OmpA (for details of preparation, see Supplementary Data), 0.5 mM for each protein, were incubated with IP antibody (Anti-GST antibody; Sungene biotech, # KM8005, China) overnight at 4°C to form antigen-antibody complex, following which complexes were bound to Protein A/G magnetic beads for 1 h at room temperature. Beads were washed twice with IP Lysis/Wash Buffer and once with purified water. The antigen/antibody complexes were eluted and collected and analyzed by western blot using Anti-GST antibody and Anti-His antibody.

### Reveres Transcription-Polymerase Chain Reaction

Bacteria were cultured to logarithmic growth phase, and the cells were collected by centrifugation at 3000 × *g* for 5 min. RNA was extracted using Bacterial RNA extraction Kit (Omega, #R6950-01). An aliquot of total RNA (0.5 μg) was reverse transcribed to cDNA using PrimeScript^TM^ RT reagent with gDNA eraser (Perfect Real Time) kit (TAKARA, #RR047A, Japan), following the manufacturer’s instructions. Diluted cDNA was subjected to real-time PCR using TB greenTM premix Ex taq II (Tli RNaseH plus) (TAKARA, #RR820A, Japan) and the RT-PCR–specific primers listed in Supplementary Table S2: *E. coli* 16S rRNA (*E. coli* 16SF and *E. coli* 16SR), *ompA* (OmpA-RTF and OmpA-RTR). Data is represented after normalization with 16S rRNA.

### Statistical Analysis

All data analysis was performed using SPSS version 19.0 (SPSS, Inc., Chicago, IL, United States). One-way analysis of variance (ANOVA) was performed on the normal distribution data. Qualitative data were analyzed using chi-square test and two-sided Fisher’s exact test. *P*-values < 0.05 were considered statistically significant.

## Results

### Increased Resistance of *E. coli* Strains to Ampicillin Is Associated With Higher Susceptibility to Phage Lysis

**FIGURE 1:**
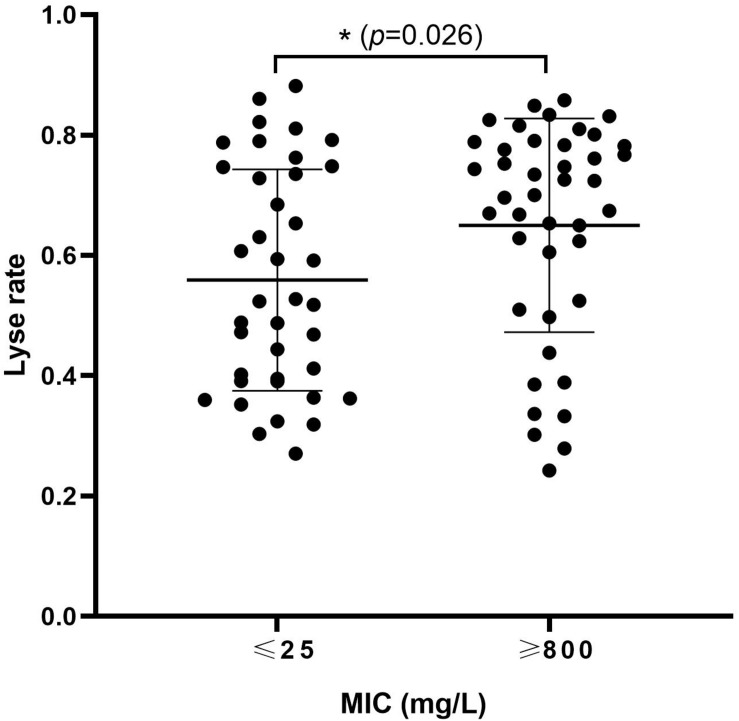
High levels of ampicillin resistance are associated with increased susceptibility of *Escherichia coli* to phage. Thirty-four *E. coli* strains and 81 phages isolated with these strains were used to investigate the relationship between ampicillin MIC and phage sensitivity of *E. coli*. Each dot: the lyse rate for a phage on its host. ^∗^0.01 < *p* < 0.05.

To study the association between ampicillin resistance and phage lysis, 34 strains of *E. coli* were used in the isolation of, and host-range determination for, a total of 81 phages (Table 1 and Supplementary Table S1). The MICs of ampicillin for these *E. coli* strains, and the lysis rates obtained using their associated phages, were determined. Bacterial strains were divided into low-resistance (<25 mg/L) comprising of 19 strains which were able to host a total of 36, and high-resistance (>800 mg/L) group comprising of 15 strains which were able to host a total of 42, of the tested phage. Furthermore, the high-resistance group showed a significantly higher phage sensitivity (*P* = 0.026) (Figure 1). These results suggested that increased ampicillin resistance may be associated with increased susceptibility of *E. coli* to phage.

**FIGURE 2:**
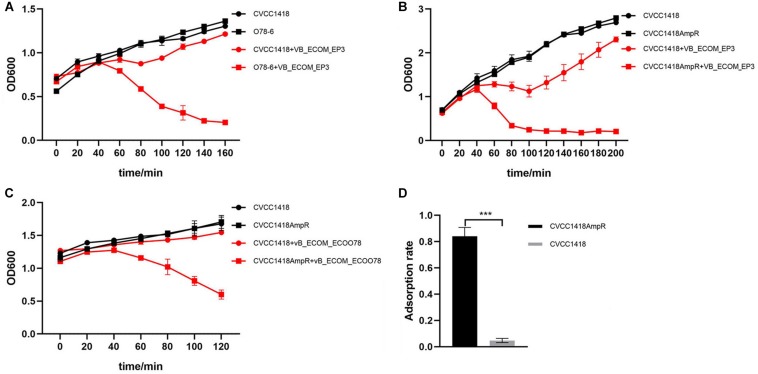
Comparison of lysis rates between serotype O78 *E. coli* isolates with high or low resistance to ampicillin. **(A)** Phage vB_EcoM-ep3 showed greater lysis of isolate O78-6 (MIC ≥ 800 mg/L) than isolate CVCC1418 (MIC ≤ 25 mg/L). **(B)** Phage vB_EcoM-ep3 showed greater lysis of CVCC1418AmpR (MIC ≥ 800 mg/L) than CVCC1418 (MIC ≤ 25 mg/L). **(C)** The related *Myoviridae* phage, vB_EcoM_ECOO78, also showed greater lysis of CVCC1418AmpR than CVCC1418. **(D)** The adsorption rate of phage vB_EcoM-ep3 onto CVCC1418AmpR was significantly higher than onto CVCC1418. ^∗∗∗^*p* < 0.01.

Four of the clinical isolates from chicken farms with high MICs of ampicillin (>800 mg/L) belong to serotype O78 and were used to isolate a total of 17 of the tested phage, including previously characterized members of the *Myoviridae* family, vB_EcoM-ep3 and vB_EcoM_ECOO78 (Lv et al., 2015; Guo et al., 2017). Full host range determination further revealed that these four strains were able to host a total of 27 phages amongst them, with individual strains hosting up to 13 different phages (Supplementary Table S1). Strain CVCC1418, also serotype O78 but with a low MIC of ampicillin (<25 mg/L), was initially used in the isolation of three phage and was further able to host vB_EcoM-ep3 and vB_EcoM_ECOO78 and four other phages, mostly those isolated using other O78 strains (Supplementary Table S1). Comparing strain O78-6 (the strain able to host the most phages) with CVCC1418 for sensitivity to lysis by phage vB_EcoM-ep3, showed that although strain CVCC1418 could host this phage, it was much less sensitive to lysis by it than the O78-6 strain (Figure 2A).

To further investigate the relationship between levels of ampicillin resistance and phage susceptibility, we generated a spontaneous mutant, CVCC1418AmpR, with higher ampicillin resistance compared to CVCC1418 and found that the mutant was much more sensitive to lysis by phage vB_EcoM-ep3 than parental strain (Figure 2B). In addition, CVCC1418AmpR was also much more sensitive than the parental strain to lysis by the related phage vB_EcoM_ECOO78 (Figure 2C). Further study found that the adsorption rate of phage vB_EcoM-ep3 was higher on strain CVCC1418AmpR than on CVCC1418 (Figure 2D). Taken together, these results demonstrate that increasing ampicillin resistance made *E. coli* strain CVCC1418 more vulnerable to the tested phage.

### Increased Ampicillin Resistance Is Associated With Differential Expression of a Number of Proteins Including AmpC and Some Outer Membrane Proteins

**FIGURE 3:**
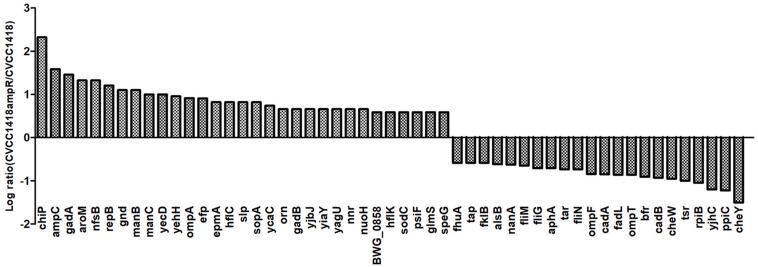
Differential protein expression in CVCC1418AmpR compared to CVCC1418. Experiments were carried out three times.

To better understand how the increased ampicillin resistance in strain CVCC1418AmpR is related to greater susceptibility to the *Myoviridae* phages vB_EcoM-ep3 and vB_EcoM_ECOO78, we compared the protein profiles of this mutant and its parental strain by iTRAQ. We identified 54 proteins that were differentially expressed, including the chromosomally encoded AmpC, which showed increased expression in the CVCC1418AmpR mutant (Figure 3). In addition, six of the differentially expressed proteins were indicated by Gene Ontology analysis to be located in the bacterial outer membrane (Supplementary Figure S1), including the porin proteins ChiP and OmpA, which were upregulated, and OmpF, which was down-regulated, in the CVCC1418AmpR mutant.

### Increased AmpC Promotes Expression of the Phage Receptor Protein OmpA

Increased AmpC expression in CVCC1418AmpR compared to the parental strain CVCC1418 was confirmed by Western blot analysis (Supplementary Figures S2A,B). However, as the CVC1418AmpR mutant was generated spontaneously by exposure to increasing concentrations of ampicillin, and proteomic analysis showed differential expression of numerous proteins in comparison to the parental strain, we sought to confirm the association between AmpC expression and phage sensitivity using genetically defined mutants.

**FIGURE 4:**
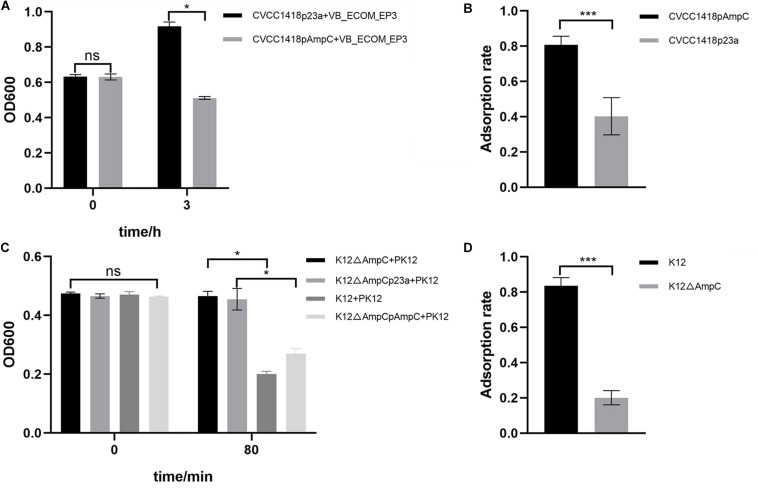
Expression of AmpC associated with increased phage adsorption and lysis. **(A)** The rate of lysis of CVCC1418pAmpC by vB_EcoM-ep3 was significantly higher than that of CVCC1418. **(B)** The adsorption rate of phage vB_EcoM-ep3 on CVCC1418pAmpC was higher than that on CVCC1418p23a. **(C)** The rate of lysis of K12△*ampC* by phage PK12 was significantly lower than that of K12 MG1655 and K12△*ampC*pAmpC while no significant difference was seen for K12△ampCp23a. **(D)** The adsorption rate of PK12 was significantly lower on K12△*ampC* than on K12 MG1655. ^∗^0.01 < *p* < 0.05; ^∗∗∗^*p* < 0.01.

Strain CVCC1418pAmpC, generated by introduction of cloned *ampC* gene on plasmid pET-23a-*ampC* (Supplementary Figure S3A), showed greater lysis by vB_EcoM-ep3, and greater adsorption of this phage, compared to the control strain containing empty vector (Figures 4A,B). As attempts to generate an *ampC* deletion mutant in strain CVCC1418 were unsuccessful, we used strain K12 MG1655 (K12) to generate an *E. coli* mutant (K12Δ*ampC*) lacking *ampC* and complemented this mutation using the pET-23a-*ampC* plasmid (Supplementary Figures S3B,C). Using phage PK12, which specifically targets strain K12 MG1655, we demonstrated that deletion of *ampC* in K12Δ*ampC* reduced lysis and adsorption by this phage compared to the parental and complemented strains, whereas introduction of the empty control vector did not restore wild-type levels of lysis or adsorption (Figures 4C,D).

The association of over-expression of the periplasmic AmpC protein with an increased phage adsorption, along the with results of iTRAQ analysis above, suggest concomitant changes in expression of phage receptor(s) located on the surface of the bacteria. In order to identify specific outer membrane receptor(s) for phage vB_EcoM-ep3, we first identified two putative PRBs of phage vB_EcoM-ep3; phage major capsid protein (ep3_0031) and putative tail fiber protein (ep3_0041) (Supplementary Table S3). BLASTx analysis of ep3_0041 revealed 99% identity with the reported tail protein-depolymerase, Dpo42 (Gene ID:40076521), of phage vB_EcoM_ECOO78. It is, therefore, speculated that ep3_0041 is also a tail-protein depolymerase, so we named it Dpo41. Since RBPs for phage are usually tail proteins, we hypothesized that Dpo41 may be the main RBP for phage vB_EcoM-ep3.

**FIGURE 5:**
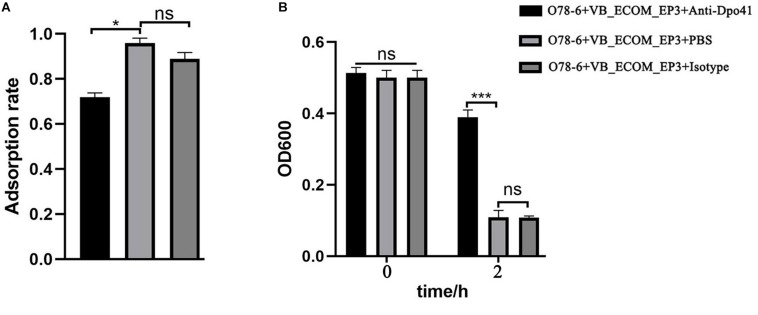
vB_EcoM-ep3 protein Dpo41 is critical for lysis of *E. coli* O78-6. Tail fiber protein Dpo41 blocked by polyclonal antibody decreased the phage adsorption efficiency **(A)** and bacteria lysis **(B)**. Anti-Dpo41: IgG obtained by immunizing rabbits with purified Dpo41, isotype: IgG obtained by immunizing rabbits with PBS. ^∗^0.01 < *p* < 0.05; ^∗∗∗^*p* < 0.01.

In order to confirm the role of Dpo41 as a RBP, we cloned, over-expressed and purified the His-tagged protein (from *E. coli* BL21-pDpo41), which was then used to generate a polyclonal rabbit antiserum (Supplementary Figure S4). Blocking Dpo41 using this antiserum significantly reduced the adsorption rate of vB_EcoM-ep3 to strain O78-6 (Figure 5A) and the lysis of this strain by the phage (Figure 5B), supporting the role of Dpo41 as RBP of phage vB_EcoM-ep3.

**FIGURE 6:**
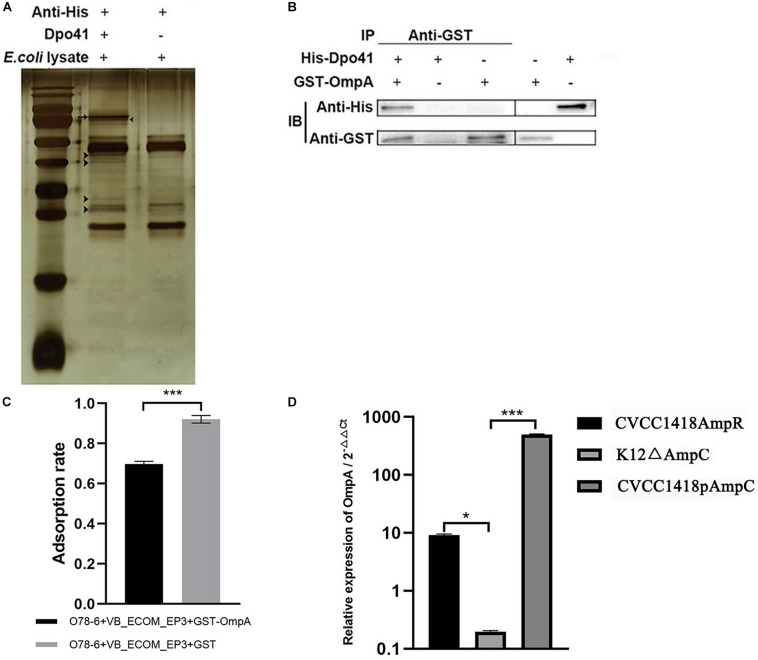
vB_EcoM-ep3 protein Dpo41 interacts with *E. coli* strain O78-6 OmpA during adsorption phase. **(A)** Identification of receptor for Dpo41 using Co-IP. Arrow: band of Dpo41, arrow head: bands for mass spectrometry. **(B)** Dpo41 bound to OmpA. The purified protein His-Dpo41 (or PBS) was incubated with purified GST-OmpA (or PBS) in the presence of Anti-GST antibody. The obtained immune complexes were subjected to CO-IP assay (see section “Materials and Methods”) and analyzed by Western blot using anti-His and Anti-GST antibodies. **(C)** OmpA pre-treatment prevented phage vB_EcoM-ep3 binding to *E. coli* strain O78-6. Phage vB_EcoM-ep3 was incubated with GST-OmpA (0.1 mg/mL) for 3 h before adsorption rate assay, the control group was treated with GST with the same amount. **(D)** Overexpression of AmpC increased the production of OmpA. The relative expression of OmpA of CVCC1418AmpR, K12Δ*ampC*, CVCC1418pAmpC were compared with that of CVCC1418, K12, CVCC1418p23a, respectively. ^∗^0.01 < *p* < 0.05; ^∗∗∗^*p* < 0.01.

Co-immunoprecipitation using the His-tagged Dpo41 protein incubated with a cell lysate of strain O78-6 identified a number of different proteins, visualized on silver stained SDS-polyacrylamide gels (Figure 6A), which were sent for mass spectrometry. The identified putative receptors were Lpp, OmpA, and Eno (Supplementary Table S4). As OmpA has previously been reported as a receptor for other *E. coli Myoviridae* (T-even) phages, we expressed a GST-OmpA fusion in *E. coli* strain BL21 (DE3) and purified this for co-immunoprecipitation analysis with His-Dpo41. The results showed that Dpo41 bound to OmpA (Figure 6B) and GST-OmpA pre-treatment prevented phage vB_EcoM-ep3 adsorption to strain O78-6 (Figure 6C). These results indicate that OmpA is a receptor for vB_EcoM-ep3.

Deletion of *ampC* significantly reduced the transcription of OmpA in *E. coli* strain K12 MG1655, whereas over-expression of AmpC in strains CVCC1418AmpR and CVCC1418pAmpC was associated with increased OmpA expression compared with strain CVCC1418 (Figure 6D). In summary, increased expression of AmpC appears to promote the production of OmpA, which in turn increases adsorption of, and lysis by, *E. coli* by phage for which OmpA is a receptor.

## Discussion

During isolation of phages using clinical isolates of *E. coli* from human and animal sources in the Changchun region of China, we noticed that those isolates with high MICs of ampicillin appeared to host a larger number of phages (Supplementary Table S1). Although only a few of the *E. coli* isolates used in this study were serotyped, we also noticed that the largest number of phages were hosted by *E. coli* originating from chicken farms belonging to serotype O78, suggesting that the common LPS in these isolates contributed to binding of a related set of phages. However, not all O78-derived phages were able to infect all of the O78 type strains, suggesting other phage receptor(s) are likely involved that differ in expression and/or accessibility amongst these isolates. That might be the reason why the difference between the two groups didn’t seem that significant (Figure 1). Furthermore, strain CVCC1418 (also serotype O78, but with a low MIC of ampicillin) hosted fewer of the O78-derived phages than the other *E. coli* of this serotype and was less susceptible to lysis by the previously characterized *Myoviridae* phages, vB_EcoM-ep3 and vB_EcoM_ECOO78 (Figure 2) (Lv et al., 2015; Guo et al., 2017). These results suggested that differences in the level of ampicillin resistance amongst O78 serotype strains may affect susceptibility to their specific phage. The 24 *E. coli* isolates that were from human sources likely belong to other serotypes, as suggested by comparatively little if any infection by O78-derived phage (Supplementary Table S1), and we did not explore the nature of the receptors bound by their associated phage in this study. These phages may bind receptors that are not affected by the level of ampicillin resistance, as suggested by some of the highly resistant isolates with lower lysis rates and the more even distribution of lysis rates for *E. coli* with low resistance (Figure 1).

We have shown that increased expression of AmpC, either via spontaneous mutation (likely in the promoter/attenuator region of the chromosomal copy of *ampC*) or by over-expression of the *ampC* gene on a plasmid, promoted decreased expression of OmpF and increased expression of OmpA in *E. coli* strain CVCC1418 (Figure 3). Lack of OmpF has previously been associated with β-lactam-resistant clinical isolates of *E. coli* hyperproducing AmpC (Martinez-Martinez et al., 2000), and loss of OmpA has previously been reported to increased sensitivity to antibiotics (Choi and Lee, 2019). Here, we have shown that the increased production of OmpA associated with increased expression of AmpC promotes the adsorption of, and lysis by, tested *Myoviridae* phages (Figures 4, 6D). We demonstrated that OmpA functions as a receptor for the tail fiber protein, Dpo41, of phage vB_EcoM-ep3, which is homologous to the tail fiber protein, Dpo42, of phage vB_EcoM_ECOO78 (Guo et al., 2017).

Our results confirm the feasibility of using phage to treat β-lactam-resistant *E. coli* infections. A previous report indicated that *Shigella flexneri* phage Sf6 uses OmpA and OmpC on the bacterial surface as receptors (Parent et al., 2014), so the phenomenon observed in this study may also be present in other Gram-negative species, which we plan to further investigate. It is possible that enhanced resistance to other antibiotics which lead to changes in expression of bacterial surface antigens, may also promote, rather than reduce, phage lysis efficiency, and these resistant bacteria can be used to screen for more efficient phages for use as biotherapeutic agents.

Our results support the combined use of phage and antibiotics to provide synergistic efficacy in treating bacterial infections, as reported by others (Oechslin et al., 2016; Ronayne et al., 2016; Wang et al., 2017). The combination of phage and antibiotics can also act synergistically to reduce bacterial density in biofilms (Akturk et al., 2019). A recent study reported that while the phage had a small effect on pathogen density on its own, it considerably increased the sensitivity of *Ralstonia solanacearum* to antibiotics produced by *Bacillus amyloliquefaciens* (Wang et al., 2017). In that study, they showed that the fitness cost of bacterial adaptation (reduced growth) was highest when the pathogen had evolved in the presence of both the phage and the competitor (Wang et al., 2017). However, the mechanism of synergism between phage and antibiotics was not fully elucidated. Another study showed that Ref endonuclease encoded by bacteriophage P1 renders *E. coli* more sensitive to the DNA-damaging antibiotic ciprofloxacin (Ronayne et al., 2016), indicating that in this case, synergy involved a mechanism other than increased phage adsorption.

## Conclusion

We investigated the mechanism by which increased AmpC expression promoted lysis of the O78 *E. coli* strain CVCC1418 by the tested phages, and identified increased production of OmpA, which we confirmed as a receptor for the *Myoviridae* phage, vB_EcoM-ep3. We further identified differential expression of other proteins following increased expression of AmpC that were not investigated as part of this study. It is possible that some of these proteins could promote lysis by other phages, as suggested by the reduced lysis of the *E. coli* K12 MG1655Δ*ampC* by phage PK12 (which does not appear to be related to the *Myoviridae* in this study), but this has yet to be explored. In general, the process of bacteria acquiring resistance to antibiotics is bound to bring about changes in expression of multiple proteins that affect bacterial metabolism and fitness, and further research is required to determine if these changes can be exploited for identification of biotherapeutic phages which can be used in combined therapies to treat bacterial infections.

## Data Availability Statement

The raw data supporting the conclusions of this article will be made available by the authors, without undue reservation, to any qualified researcher.

## Ethics Statement

New Zealand white rabbits (female, 1.3–1.6 kg) were purchased from Changchun Yisi Experimental Animal Technology, Co., Ltd. (Changchun, China). All animal managements and experiments were strictly abided by the Regulations for the Administration of Affairs Concerning Experimental Animals approved by the State Council of the People’s Republic of China (1.11.1988) and approved by the Animal Welfare and Research Ethics Committee at Jilin University.

## Author Contributions

SW, JB, BY, and LY drafted the main manuscript. MD performed the data analysis. SW, BY, LY, and ZG planned and performed the experiments. SW, JG, CD, XF, WH, YA, CS, and LL were responsible for experimental design. SW, GY, DH, and LL were responsible for guiding and supporting the experiments and manuscript revisions.

## Conflict of Interest

The authors declare that the research was conducted in the absence of any commercial or financial relationships that could be construed as a potential conflict of interest.

## Footnotes

1 https://www.genewiz.com.cn/
