# Supplementary material for: Overexpression of AmpC Promotes Bacteriophage Lysis of Ampicillin-Resistant Escherichia coli
Source: Front Microbiol. 2020 Jan 8;10:2973. doi: 10.3389/fmicb.2019.02973 (PMC6960117; doi:10.3389/fmicb.2019.02973)
Supplement: Supplementary file 1 [file Data_Sheet_1.docx]

**Supplemental materials**

# Methods

# Clone and expression of GST-OmpA and His-Dpo41

Genome of E. coli strain CVCC1418 was used as template and OmpAF / OmpAR were used as primers to amplify OmpA. Enzyme digestion was performed using endonucleases EcoRI and SmaI. Obtained *ompa* gene was introduced into the vector pGEX-6P-1. The constructed plasmid was introduced into BL21 (DE3) by heat shock and screened using LB plate containing ampicillin (100 μg/mL). Positive clone was induced to express OmpA using IPTG (0.8 mM), and expressed proteins were purified using GenScript Glutathione Resin (GenScript, #L00206, China) according to the manufacturer's instructions. For clone and expression of Dpo41, genome of VB_ECOM_EP3 was used as template and Dpo41F/ Dpo41R were used as primers. Enzyme digestion was performed using the endonucleases BamHⅠ and XhoⅠ. Obtained *Dpo41* gene was introduced into the vector pET-28a and transformed into BL21 (DE3) by heat shock and screened using LB plate containing kanamycin (34 μg/mL). Positive clone was induced to express Dpo41 using IPTG (0.8 mM), and expressed proteins were purified using High Affinity Ni-Resin (GenScript, #L00250, China) according to the manufacturer's instructions.

# Production of anti-AmpC and anti-Dpo41serum

On day 0, purified protein was mixed in equal volumes with complete Freund's adjuvant and emulsified and subcutaneously injected into rabbit (1.3–1.6 kg), 500 μg of protein per rabbit. On the 7th day, the second immunization was carried out. Purified protein was mixed in equal volumes with incomplete Freund's adjuvant and emulsified and subcutaneously injected into rabbit, 200 μg of protein per rabbit. The third immunization was performed on the 21st day, and the operation was the same as the second immunization. On the 28th day, blood was collected from the rabbit heart and the rabbit was euthanized. Serum antibody titers were measured using an indirect ELISA.

# Figures


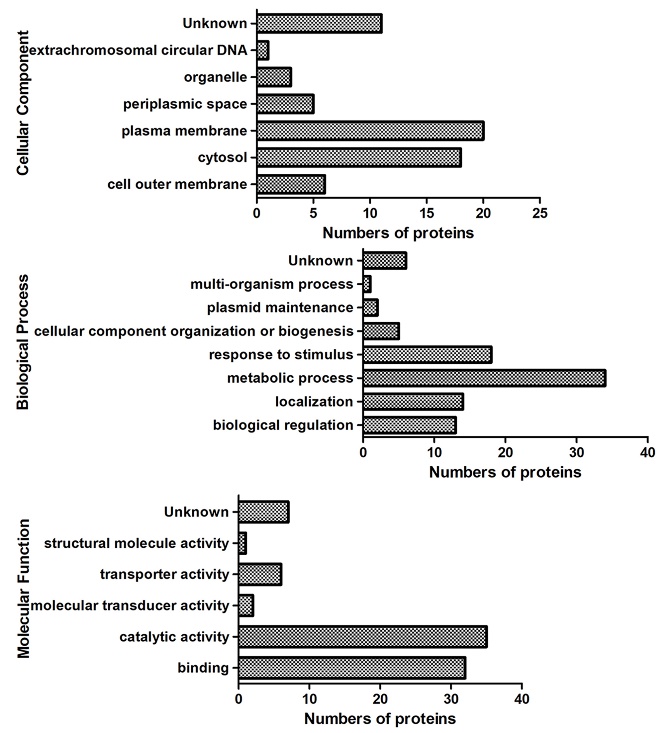


**Figure S1. Cellular component、biological process、molecular function of proteins with differential expression.**

**
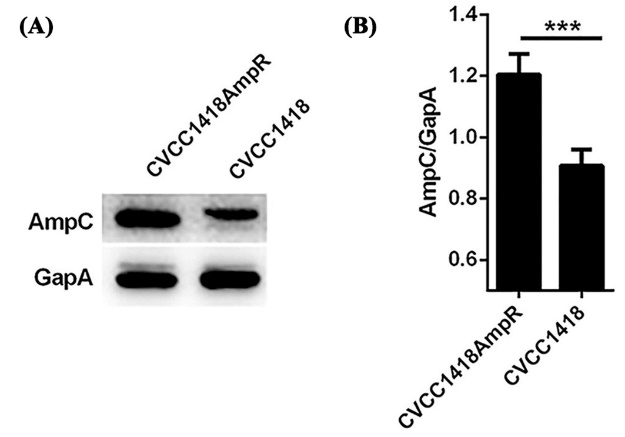
**

**Figure S2. AmpC was increased in CVCC1418AmpR. (A)** Western blot showing relative amounts of AmpC in CVCC1418AmpR and CVCC1418. **(B)** Quantification of the relative expression of AmpC from (**A)**.


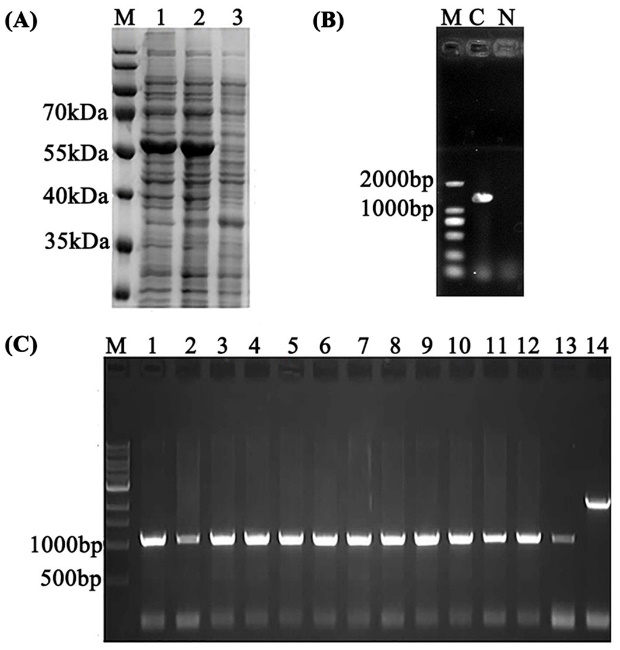


**Figure S3. Validation of AmpC expression or knockout. (A)** The small-scale expression of AmpC in CVCC1418pAmpC. Lane: 1and 2: whole cell; Lane: 3, negative control. **(B)** PCR validation of knockout of AmpC using primers pUAmpC1/pDAmpC2. Lanes 1, 2, 3, 4, 5, 6, 7, 8, 9, 10, 11, 12, 13 were amplified using genomic DNA extracted from strains cured of the TargetT-ΔAmpC as template. Lane 14 was amplified using genomic DNA derived from untreated K12 MG1655 as template. **(C)** PCR validation of complementation of AmpC into K12ΔAmpC. Lane C was amplified using plasmid extracted from strains transferred with pET-23a-AmpC as template. Lane 14 was amplified using genomic DNA from untreated K12ΔAmpC as template.

**
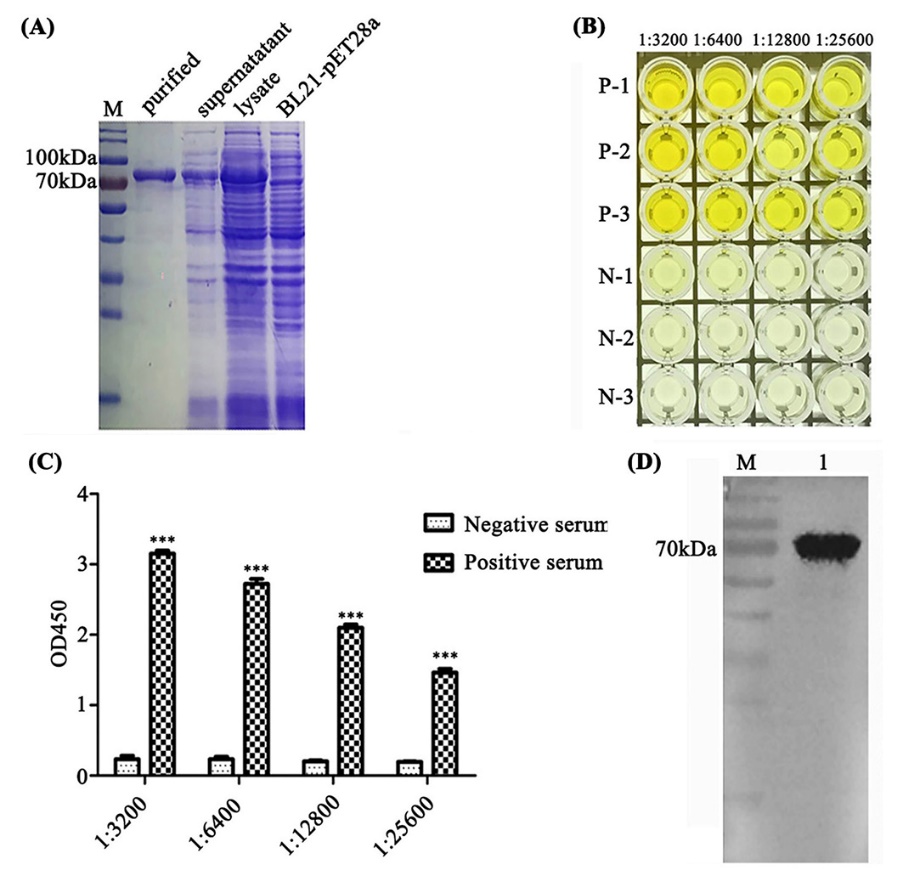
**

**Figure S4. Preparation of Dpo41 and its polyclonal antibody .(A)** The expression and purification of Dpo41. Purified: purified Dpo41; Supernatant: supernatant of induced *E. coli* BL21-pDpo41 after ultrasonic decomposition; Lysate: induced *E. coli* BL21-pDpo41 after ultrasonic decomposition; BL21-pET28a: *E. coli* BL21-pET28a cells. **(B)** and **(C)** The titer of Dpo41 polyclonal antibody was measured by ELISA and the titer was 1:25600. **(D)** The purity of Dpo41 polyclonal antibody was detected by WB. Lane 1 was O78-6 bacterial lysate.

# Tables

**Table S1. Host spectra of phages.**

| *E. coli*  strains  Phages | O78-5 | CVCC1418AmpR | CVCC1418 | O78-3 | O78-6 | CC11 | F23 | ATCC25922 | K12MG1655 | BL21(DE3) | HXM | LSZ | CZ | ZLH | WZ | SGF | ZDZ | LHY | 84 | YFX | TC | MLD | DQQ | SWD | TSQ | ZDY | GYP | MYL | ZZJ | BDY | PJ | YXM | ZX | WHL |
| --- | --- | --- | --- | --- | --- | --- | --- | --- | --- | --- | --- | --- | --- | --- | --- | --- | --- | --- | --- | --- | --- | --- | --- | --- | --- | --- | --- | --- | --- | --- | --- | --- | --- | --- |
| PO78-5-1 | ● | Y | Y | ○ | ○ | ○ | ○ | ○ | ○ | Y | ○ | ○ | ○ | ○ | ○ | ○ | ○ | ○ | ○ | ○ | ○ | ○ | ○ | ○ | ○ | ○ | ○ | ○ | ○ | ○ | ○ | ○ | ○ | ○ |
| PO78-5-2 | ● | Y | ○ | ○ | ○ | ○ | ○ | ○ | ○ | ○ | ○ | ○ | ○ | ○ | ○ | ○ | ○ | ○ | ○ | ○ | ○ | ○ | ○ | ○ | ○ | ○ | ○ | ○ | ○ | ○ | ○ | ○ | ○ | ○ |
| PAMPR-1 | ○ | ● | ○ | ○ | ○ | ○ | ○ | ○ | ○ | ○ | ○ | ○ | ○ | ○ | ○ | ○ | ○ | ○ | ○ | ○ | ○ | ○ | ○ | ○ | ○ | ○ | ○ | ○ | ○ | ○ | ○ | ○ | ○ | ○ |
| PAMPR-2 | Y | ● | ○ | Y | ○ | ○ | ○ | ○ | ○ | ○ | Y | ○ | ○ | ○ | ○ | Y | ○ | ○ | ○ | ○ | ○ | ○ | ○ | ○ | ○ | ○ | ○ | ○ | ○ | ○ | ○ | ○ | ○ | ○ |
| PAMPR-3 | ○ | ● | ○ | Y | Y | Y | ○ | ○ | ○ | ○ | ○ | ○ | ○ | ○ | ○ | ○ | ○ | ○ | ○ | ○ | ○ | ○ | ○ | ○ | ○ | ○ | ○ | ○ | ○ | ○ | ○ | ○ | ○ | ○ |
| PAMPR-4 | ○ | ● | Y | ○ | ○ | ○ | ○ | ○ | ○ | ○ | ○ | ○ | ○ | ○ | ○ | ○ | ○ | ○ | ○ | ○ | ○ | ○ | ○ | Y | ○ | ○ | ○ | ○ | ○ | ○ | ○ | ○ | ○ | ○ |
| P1418-1 | ○ | Y | ● | ○ | Y | ○ | ○ | ○ | ○ | ○ | ○ | ○ | ○ | ○ | ○ | ○ | ○ | ○ | ○ | ○ | ○ | ○ | ○ | ○ | ○ | ○ | ○ | ○ | ○ | ○ | ○ | ○ | ○ | ○ |
| P1418-2 | ○ | ○ | ● | Y | ○ | ○ | ○ | ○ | ○ | ○ | ○ | ○ | ○ | ○ | ○ | ○ | ○ | ○ | ○ | ○ | ○ | ○ | ○ | ○ | ○ | ○ | ○ | ○ | ○ | ○ | ○ | ○ | ○ | ○ |
| P1418-3 | ○ | Y | ● | ○ | ○ | ○ | ○ | ○ | ○ | ○ | ○ | ○ | ○ | ○ | ○ | ○ | ○ | ○ | ○ | ○ | ○ | ○ | ○ | ○ | ○ | ○ | ○ | ○ | ○ | ○ | ○ | ○ | ○ | ○ |
| VB_ECOM_ECOO78 | ○ | ○ | Y | ● | Y | ○ | Y | ○ | ○ | ○ | ○ | ○ | ○ | ○ | ○ | ○ | ○ | ○ | ○ | ○ | ○ | ○ | ○ | ○ | ○ | ○ | ○ | ○ | ○ | ○ | ○ | ○ | ○ | ○ |
| PO78-3-2 | ○ | Y | ○ | ● | ○ | ○ | ○ | ○ | ○ | ○ | ○ | ○ | ○ | ○ | ○ | ○ | ○ | ○ | ○ | ○ | ○ | ○ | ○ | ○ | ○ | ○ | ○ | ○ | ○ | Y | ○ | ○ | ○ | ○ |
| PO78-3-3 | ○ | ○ | ○ | ● | ○ | ○ | ○ | ○ | ○ | ○ | ○ | ○ | ○ | ○ | ○ | ○ | ○ | ○ | ○ | ○ | ○ | ○ | ○ | ○ | ○ | ○ | ○ | ○ | ○ | ○ | ○ | ○ | ○ | ○ |
| PO78-3-4 | ○ | ○ | ○ | ● | Y | ○ | ○ | ○ | ○ | ○ | ○ | ○ | ○ | ○ | ○ | ○ | ○ | ○ | ○ | ○ | ○ | ○ | ○ | ○ | ○ | ○ | ○ | ○ | ○ | ○ | ○ | ○ | ○ | ○ |
| PO78-6-2 | ○ | Y | ○ | ○ | ● | ○ | ○ | ○ | ○ | ○ | ○ | ○ | ○ | ○ | ○ | ○ | ○ | ○ | ○ | ○ | ○ | Y | ○ | ○ | ○ | ○ | ○ | ○ | ○ | ○ | ○ | ○ | ○ | ○ |
| PO78-6-3 | ○ | Y | ○ | ○ | ● | ○ | Y | ○ | ○ | ○ | ○ | ○ | ○ | ○ | ○ | ○ | ○ | ○ | ○ | ○ | ○ | ○ | ○ | ○ | ○ | ○ | ○ | ○ | ○ | ○ | ○ | ○ | ○ | ○ |
| PO78-6-4 | ○ | Y | ○ | ○ | ● | ○ | ○ | ○ | ○ | ○ | ○ | ○ | ○ | ○ | ○ | ○ | ○ | ○ | ○ | ○ | ○ | ○ | ○ | ○ | ○ | ○ | ○ | ○ | ○ | ○ | ○ | ○ | ○ | ○ |
| PO78-6-5 | ○ | ○ | Y | ○ | ● | ○ | ○ | ○ | ○ | ○ | ○ | ○ | ○ | ○ | ○ | ○ | ○ | ○ | ○ | Y | ○ | ○ | ○ | ○ | ○ | ○ | ○ | ○ | ○ | ○ | ○ | ○ | ○ | ○ |
| PO78-6-6 | ○ | ○ | ○ | ○ | ● | ○ | ○ | ○ | ○ | ○ | ○ | ○ | ○ | ○ | ○ | ○ | ○ | ○ | ○ | ○ | ○ | ○ | ○ | ○ | ○ | ○ | ○ | ○ | ○ | ○ | ○ | ○ | ○ | ○ |
| VB_ECOM_EP3 | Y | Y | Y | Y | Y | ● | Y | ○ | ○ | ○ | ○ | ○ | ○ | ○ | ○ | Y | ○ | ○ | ○ | Y | ○ | ○ | ○ | ○ | ○ | ○ | ○ | ○ | ○ | ○ | ○ | ○ | ○ | ○ |
| PCC11-1 | ○ | ○ | ○ | ○ | Y | ● | ○ | ○ | ○ | ○ | ○ | ○ | ○ | ○ | ○ | ○ | ○ | ○ | ○ | ○ | ○ | ○ | ○ | ○ | ○ | ○ | ○ | ○ | ○ | ○ | ○ | ○ | ○ | ○ |
| PCC11-2 | Y | ○ | ○ | ○ | ○ | ● | ○ | ○ | ○ | ○ | ○ | ○ | ○ | ○ | ○ | ○ | ○ | ○ | ○ | ○ | ○ | ○ | ○ | ○ | ○ | ○ | ○ | ○ | ○ | ○ | ○ | ○ | ○ | ○ |
| PCC11-3 | Y | Y | ○ | ○ | ○ | ● | ○ | ○ | ○ | ○ | ○ | ○ | ○ | ○ | ○ | ○ | ○ | ○ | ○ | ○ | ○ | ○ | ○ | ○ | ○ | ○ | ○ | ○ | ○ | ○ | ○ | ○ | ○ | ○ |
| PCC11-4 | ○ | ○ | ○ | Y | ○ | ● | ○ | ○ | ○ | ○ | ○ | ○ | ○ | ○ | ○ | ○ | ○ | ○ | ○ | ○ | ○ | ○ | ○ | ○ | ○ | Y | ○ | ○ | ○ | ○ | ○ | ○ | Y | ○ |
| PCC11-5 | ○ | ○ | ○ | Y | ○ | ● | ○ | ○ | ○ | ○ | ○ | ○ | ○ | ○ | ○ | ○ | ○ | ○ | ○ | ○ | ○ | ○ | ○ | ○ | ○ | ○ | ○ | ○ | ○ | ○ | ○ | ○ | ○ | ○ |
| PF23-1 | ○ | ○ | ○ | ○ | Y | ○ | ● | ○ | ○ | ○ | ○ | ○ | ○ | ○ | ○ | ○ | ○ | ○ | ○ | ○ | ○ | ○ | ○ | ○ | ○ | ○ | ○ | Y | Y | ○ | ○ | ○ | ○ | ○ |
| PF23-2 | ○ | ○ | ○ | ○ | ○ | ○ | ● | ○ | ○ | ○ | ○ | ○ | ○ | ○ | ○ | ○ | ○ | ○ | ○ | ○ | ○ | ○ | ○ | ○ | ○ | ○ | ○ | ○ | ○ | ○ | ○ | ○ | ○ | ○ |
| PF23-3 | ○ | ○ | ○ | ○ | ○ | ○ | ● | ○ | ○ | ○ | ○ | ○ | ○ | ○ | ○ | ○ | ○ | ○ | Y | ○ | ○ | ○ | ○ | ○ | ○ | ○ | ○ | ○ | ○ | ○ | ○ | ○ | ○ | ○ |
| PF23-4 | ○ | ○ | ○ | ○ | ○ | ○ | ● | ○ | ○ | ○ | ○ | ○ | ○ | ○ | ○ | ○ | ○ | ○ | ○ | ○ | ○ | ○ | ○ | ○ | ○ | ○ | ○ | ○ | ○ | ○ | ○ | ○ | ○ | ○ |
| JDP1 | ○ | ○ | ○ | ○ | ○ | ○ | ○ | ● | ○ | ○ | ○ | ○ | ○ | ○ | ○ | ○ | ○ | ○ | ○ | ○ | ○ | ○ | ○ | ○ | ○ | ○ | ○ | ○ | ○ | ○ | ○ | ○ | ○ | ○ |
| RB | ○ | ○ | ○ | ○ | ○ | ○ | ○ | ● | ○ | ○ | ○ | ○ | ○ | ○ | ○ | ○ | ○ | ○ | ○ | ○ | ○ | ○ | ○ | ○ | ○ | ○ | ○ | ○ | ○ | ○ | ○ | ○ | ○ | ○ |
| RS | ○ | ○ | ○ | ○ | ○ | ○ | ○ | ● | ○ | ○ | ○ | ○ | ○ | ○ | ○ | ○ | ○ | ○ | ○ | ○ | ○ | ○ | ○ | ○ | ○ | ○ | ○ | ○ | ○ | ○ | ○ | ○ | ○ | ○ |
| PK12 | ○ | ○ | ○ | ○ | ○ | ○ | ○ | ○ | ● | ○ | ○ | ○ | ○ | ○ | ○ | ○ | ○ | ○ | ○ | ○ | ○ | ○ | ○ | ○ | ○ | ○ | ○ | ○ | ○ | ○ | ○ | ○ | ○ | ○ |
| PK12-2 | ○ | ○ | ○ | ○ | ○ | ○ | ○ | ○ | ● | ○ | ○ | ○ | ○ | ○ | ○ | ○ | ○ | ○ | ○ | ○ | ○ | ○ | ○ | ○ | ○ | ○ | ○ | ○ | ○ | ○ | ○ | ○ | ○ | ○ |
| PBL21-1 | ○ | ○ | ○ | ○ | ○ | ○ | ○ | ○ | ○ | ● | ○ | ○ | ○ | ○ | ○ | ○ | ○ | ○ | ○ | Y | ○ | ○ | ○ | ○ | ○ | ○ | ○ | ○ | ○ | ○ | ○ | ○ | ○ | ○ |
| PBL21-2 | ○ | ○ | ○ | ○ | ○ | ○ | ○ | ○ | ○ | ● | ○ | ○ | ○ | ○ | ○ | ○ | ○ | ○ | ○ | ○ | ○ | ○ | ○ | ○ | ○ | ○ | ○ | ○ | ○ | ○ | ○ | ○ | ○ | ○ |
| PHXM | ○ | Y | ○ | ○ | ○ | ○ | ○ | ○ | ○ | ○ | ● | ○ | ○ | ○ | ○ | ○ | ○ | ○ | ○ | ○ | ○ | ○ | Y | ○ | ○ | ○ | ○ | ○ | ○ | ○ | ○ | ○ | ○ | ○ |
| PLSZ | ○ | ○ | ○ | ○ | ○ | ○ | ○ | ○ | ○ | ○ | ○ | ● | ○ | ○ | ○ | ○ | ○ | ○ | ○ | ○ | ○ | ○ | ○ | ○ | ○ | ○ | ○ | ○ | ○ | ○ | ○ | ○ | ○ | ○ |
| PCZ-1 | ○ | ○ | ○ | ○ | ○ | ○ | ○ | ○ | ○ | ○ | ○ | ○ | ● | ○ | ○ | ○ | ○ | ○ | ○ | ○ | ○ | ○ | ○ | ○ | ○ | ○ | ○ | ○ | ○ | ○ | ○ | ○ | ○ | ○ |
| PCZ-2 | ○ | ○ | ○ | ○ | ○ | ○ | ○ | ○ | ○ | ○ | ○ | ○ | ● | ○ | ○ | ○ | ○ | ○ | ○ | ○ | ○ | ○ | ○ | ○ | ○ | ○ | ○ | ○ | ○ | ○ | ○ | ○ | ○ | ○ |
| PCZ-3 | ○ | ○ | ○ | ○ | ○ | ○ | ○ | ○ | ○ | ○ | ○ | ○ | ● | ○ | ○ | ○ | ○ | ○ | ○ | Y | ○ | ○ | ○ | ○ | ○ | ○ | ○ | ○ | ○ | ○ | ○ | ○ | ○ | ○ |
| PZLH-1 | ○ | Y | ○ | ○ | ○ | ○ | ○ | ○ | ○ | ○ | ○ | ○ | ○ | ● | ○ | ○ | ○ | ○ | ○ | ○ | ○ | ○ | ○ | ○ | ○ | ○ | ○ | ○ | ○ | ○ | ○ | ○ | ○ | ○ |
| PZLH-2 | ○ | ○ | ○ | ○ | ○ | ○ | ○ | ○ | ○ | ○ | ○ | ○ | ○ | ● | ○ | ○ | ○ | ○ | ○ | ○ | ○ | ○ | ○ | ○ | ○ | ○ | ○ | ○ | Y | ○ | ○ | ○ | ○ | ○ |
| PZLH-3 | Y | ○ | ○ | ○ | ○ | ○ | ○ | ○ | ○ | ○ | ○ | ○ | ○ | ● | ○ | ○ | ○ | ○ | ○ | ○ | ○ | ○ | ○ | ○ | ○ | ○ | ○ | ○ | ○ | ○ | ○ | ○ | ○ | ○ |
| PWZ-1 | ○ | ○ | ○ | ○ | ○ | ○ | ○ | Y | ○ | ○ | ○ | ○ | ○ | ○ | ● | ○ | ○ | ○ | ○ | ○ | ○ | ○ | ○ | ○ | ○ | ○ | ○ | ○ | ○ | ○ | ○ | ○ | ○ | ○ |
| PWZ-2 | Y | ○ | ○ | ○ | ○ | ○ | ○ | ○ | ○ | ○ | ○ | ○ | ○ | ○ | ● | ○ | ○ | ○ | ○ | ○ | ○ | ○ | ○ | ○ | ○ | ○ | ○ | ○ | ○ | ○ | ○ | ○ | ○ | ○ |
| PWZ-3 | ○ | ○ | ○ | ○ | ○ | ○ | ○ | ○ | ○ | ○ | ○ | ○ | ○ | ○ | ● | ○ | ○ | ○ | ○ | ○ | ○ | ○ | ○ | ○ | ○ | ○ | ○ | ○ | ○ | ○ | ○ | ○ | ○ | ○ |
| PWZ-4 | ○ | ○ | ○ | ○ | ○ | ○ | ○ | ○ | ○ | ○ | ○ | ○ | ○ | ○ | ● | ○ | ○ | ○ | ○ | ○ | ○ | ○ | ○ | ○ | ○ | ○ | ○ | Y | Y | ○ | ○ | ○ | ○ | ○ |
| PSGF | ○ | ○ | ○ | ○ | ○ | ○ | ○ | ○ | ○ | ○ | ○ | ○ | Y | ○ | ○ | ● | ○ | ○ | ○ | ○ | ○ | ○ | ○ | ○ | ○ | ○ | ○ | ○ | ○ | ○ | ○ | ○ | ○ | ○ |
| PZDZ-1 | ○ | ○ | ○ | ○ | Y | ○ | ○ | ○ | ○ | ○ | ○ | Y | ○ | ○ | ○ | ○ | ● | ○ | ○ | ○ | ○ | ○ | ○ | ○ | ○ | ○ | ○ | ○ | ○ | ○ | ○ | ○ | ○ | ○ |
| PZDZ-2 | ○ | ○ | ○ | ○ | ○ | ○ | ○ | ○ | ○ | ○ | ○ | ○ | ○ | ○ | ○ | ○ | ● | ○ | ○ | ○ | ○ | ○ | ○ | ○ | ○ | ○ | ○ | ○ | ○ | ○ | ○ | ○ | ○ | ○ |
| PZDZ-3 | ○ | ○ | ○ | ○ | ○ | ○ | ○ | ○ | ○ | ○ | ○ | ○ | ○ | ○ | ○ | ○ | ● | ○ | ○ | ○ | ○ | ○ | ○ | ○ | ○ | ○ | ○ | ○ | ○ | ○ | ○ | ○ | ○ | ○ |
| PLHY | ○ | ○ | Y | ○ | ○ | ○ | ○ | ○ | ○ | ○ | ○ | ○ | ○ | ○ | ○ | ○ | ○ | ● | ○ | ○ | ○ | ○ | ○ | ○ | ○ | ○ | ○ | ○ | ○ | ○ | ○ | ○ | ○ | ○ |
| P84 | ○ | ○ | ○ | ○ | ○ | ○ | Y | ○ | ○ | ○ | ○ | ○ | ○ | ○ | ○ | ○ | ○ | ○ | ● | ○ | ○ | ○ | ○ | ○ | Y | ○ | ○ | ○ | ○ | ○ | ○ | ○ | ○ | ○ |
| PYFX | ○ | ○ | ○ | ○ | ○ | ○ | ○ | ○ | ○ | ○ | ○ | ○ | ○ | ○ | ○ | ○ | ○ | ○ | ○ | ● | ○ | ○ | ○ | ○ | ○ | ○ | ○ | ○ | ○ | ○ | ○ | ○ | ○ | ○ |
| PTC-1 | ○ | ○ | ○ | ○ | ○ | ○ | ○ | ○ | ○ | ○ | Y | ○ | ○ | ○ | ○ | ○ | ○ | ○ | ○ | ○ | ● | ○ | ○ | ○ | ○ | ○ | ○ | ○ | ○ | ○ | ○ | ○ | ○ | ○ |
| PTC-2 | ○ | ○ | ○ | ○ | ○ | ○ | ○ | ○ | ○ | ○ | ○ | ○ | ○ | ○ | ○ | ○ | ○ | ○ | ○ | ○ | ● | ○ | ○ | ○ | ○ | ○ | ○ | ○ | ○ | ○ | ○ | ○ | ○ | ○ |
| PMLD-1 | ○ | ○ | ○ | ○ | ○ | ○ | ○ | ○ | ○ | ○ | ○ | ○ | ○ | ○ | ○ | ○ | ○ | ○ | ○ | ○ | ○ | ● | ○ | ○ | ○ | ○ | ○ | ○ | ○ | ○ | ○ | ○ | ○ | ○ |
| PMLD-2 | ○ | ○ | ○ | ○ | ○ | ○ | ○ | ○ | ○ | ○ | ○ | ○ | ○ | ○ | ○ | ○ | ○ | ○ | ○ | ○ | ○ | ● | ○ | ○ | ○ | ○ | ○ | ○ | ○ | ○ | ○ | ○ | ○ | ○ |
| PDQQ-1 | ○ | ○ | ○ | ○ | ○ | ○ | ○ | ○ | ○ | ○ | ○ | ○ | ○ | ○ | ○ | ○ | ○ | ○ | ○ | ○ | ○ | ○ | ● | ○ | ○ | ○ | ○ | ○ | ○ | ○ | ○ | ○ | ○ | ○ |
| PDQQ-2 | ○ | ○ | ○ | ○ | ○ | ○ | ○ | ○ | ○ | ○ | ○ | ○ | ○ | Y | ○ | ○ | ○ | Y | ○ | ○ | ○ | ○ | ● | ○ | ○ | ○ | ○ | ○ | ○ | ○ | ○ | ○ | ○ | ○ |
| PDQQ-3 | ○ | ○ | ○ | ○ | ○ | ○ | ○ | ○ | ○ | ○ | ○ | ○ | ○ | ○ | ○ | ○ | ○ | ○ | ○ | ○ | ○ | ○ | ● | Y | ○ | ○ | ○ | ○ | ○ | ○ | ○ | ○ | ○ | ○ |
| PSWD-1 | ○ | ○ | ○ | ○ | ○ | ○ | ○ | Y | Y | ○ | ○ | ○ | ○ | ○ | ○ | ○ | ○ | ○ | ○ | ○ | ○ | ○ | ○ | ● | ○ | ○ | ○ | ○ | ○ | ○ | ○ | ○ | ○ | ○ |
| PSWD-2 | ○ | ○ | ○ | ○ | ○ | ○ | ○ | ○ | ○ | ○ | ○ | ○ | ○ | ○ | ○ | ○ | ○ | ○ | ○ | ○ | ○ | Y | ○ | ● | ○ | ○ | ○ | ○ | ○ | ○ | ○ | ○ | ○ | ○ |
| PTSQ | ○ | ○ | ○ | ○ | ○ | ○ | ○ | ○ | ○ | ○ | ○ | ○ | ○ | ○ | ○ | ○ | ○ | ○ | ○ | ○ | ○ | ○ | ○ | ○ | ● | ○ | ○ | ○ | ○ | ○ | ○ | ○ | ○ | ○ |
| PZDY | ○ | ○ | ○ | ○ | ○ | ○ | ○ | ○ | ○ | ○ | ○ | ○ | ○ | ○ | ○ | ○ | ○ | ○ | ○ | ○ | ○ | ○ | ○ | ○ | ○ | ● | ○ | ○ | ○ | ○ | ○ | ○ | ○ | ○ |
| PGYP-1 | ○ | ○ | ○ | ○ | ○ | ○ | ○ | ○ | ○ | ○ | ○ | ○ | ○ | ○ | ○ | ○ | Y | ○ | ○ | ○ | ○ | ○ | ○ | ○ | ○ | ○ | ● | ○ | ○ | ○ | ○ | ○ | ○ | ○ |
| PGYP-2 | ○ | ○ | ○ | ○ | ○ | ○ | Y | ○ | ○ | ○ | ○ | ○ | ○ | ○ | ○ | ○ | ○ | ○ | ○ | ○ | ○ | ○ | ○ | ○ | ○ | ○ | ● | ○ | ○ | ○ | ○ | ○ | ○ | ○ |
| PMYL-1 | ○ | ○ | ○ | ○ | ○ | ○ | ○ | ○ | ○ | ○ | ○ | ○ | ○ | ○ | ○ | ○ | ○ | ○ | ○ | ○ | ○ | ○ | ○ | ○ | ○ | ○ | ○ | ● | ○ | ○ | ○ | ○ | ○ | ○ |
| PMYL-2 | ○ | ○ | ○ | ○ | ○ | ○ | ○ | ○ | ○ | ○ | ○ | ○ | ○ | ○ | ○ | ○ | ○ | ○ | ○ | ○ | ○ | ○ | ○ | ○ | ○ | ○ | ○ | ● | ○ | ○ | ○ | ○ | ○ | ○ |
| PZZJ-1 | ○ | ○ | ○ | ○ | ○ | ○ | ○ | ○ | ○ | ○ | ○ | ○ | ○ | Y | ○ | ○ | ○ | ○ | ○ | ○ | ○ | ○ | ○ | ○ | ○ | ○ | ○ | ○ | ● | ○ | ○ | ○ | ○ | ○ |
| PZZJ-2 | ○ | ○ | ○ | ○ | ○ | ○ | ○ | ○ | ○ | ○ | ○ | ○ | ○ | ○ | ○ | ○ | ○ | ○ | Y | ○ | ○ | ○ | ○ | ○ | ○ | Y | ○ | ○ | ● | ○ | ○ | ○ | ○ | ○ |
| PBDY | ○ | ○ | ○ | ○ | ○ | ○ | ○ | ○ | ○ | ○ | ○ | ○ | ○ | ○ | ○ | ○ | ○ | ○ | ○ | ○ | ○ | ○ | ○ | ○ | ○ | Y | ○ | ○ | ○ | ● | ○ | ○ | ○ | ○ |
| PPJ-1 | Y | ○ | ○ | ○ | ○ | ○ | ○ | ○ | ○ | ○ | ○ | ○ | ○ | ○ | ○ | ○ | ○ | ○ | ○ | ○ | ○ | ○ | ○ | ○ | ○ | ○ | ○ | ○ | ○ | ○ | ● | ○ | ○ | ○ |
| PPJ-2 | ○ | ○ | ○ | ○ | ○ | ○ | ○ | ○ | ○ | ○ | ○ | ○ | ○ | ○ | ○ | ○ | ○ | ○ | ○ | ○ | ○ | ○ | ○ | ○ | Y | ○ | ○ | ○ | ○ | ○ | ● | ○ | ○ | ○ |
| PPJ-3 | ○ | ○ | ○ | ○ | ○ | ○ | ○ | ○ | ○ | ○ | ○ | ○ | ○ | ○ | ○ | ○ | ○ | ○ | ○ | ○ | ○ | ○ | ○ | ○ | ○ | ○ | ○ | ○ | ○ | ○ | ● | ○ | ○ | ○ |
| PPJ-4 | ○ | ○ | ○ | ○ | ○ | ○ | Y | ○ | ○ | ○ | ○ | ○ | ○ | ○ | ○ | ○ | ○ | ○ | ○ | ○ | ○ | ○ | ○ | ○ | ○ | ○ | ○ | ○ | ○ | ○ | ● | ○ | ○ | ○ |
| PYXM | ○ | ○ | ○ | ○ | ○ | ○ | ○ | ○ | ○ | ○ | ○ | ○ | ○ | ○ | ○ | ○ | ○ | ○ | ○ | ○ | ○ | ○ | ○ | ○ | ○ | ○ | ○ | ○ | ○ | ○ | ○ | ● | ○ | ○ |
| PZX | ○ | ○ | ○ | ○ | ○ | ○ | ○ | ○ | ○ | ○ | ○ | ○ | ○ | ○ | ○ | ○ | ○ | ○ | ○ | ○ | ○ | ○ | ○ | ○ | ○ | ○ | ○ | ○ | ○ | ○ | ○ | ○ | ● | ○ |
| PWHL-1 | ○ | ○ | ○ | ○ | ○ | ○ | ○ | ○ | ○ | ○ | ○ | ○ | ○ | ○ | ○ | ○ | ○ | ○ | ○ | ○ | ○ | ○ | ○ | ○ | ○ | ○ | Y | ○ | ○ | ○ | ○ | ○ | ○ | ● |
| PWHL-2 | Y | ○ | ○ | ○ | ○ | ○ | ○ | ○ | ○ | ○ | ○ | ○ | ○ | ○ | ○ | ○ | ○ | ○ | ○ | ○ | ○ | ○ | ○ | ○ | ○ | ○ | Y | ○ | ○ | ○ | ○ | ○ | ○ | ● |
| PWHL-3 | ○ | ○ | ○ | ○ | ○ | ○ | ○ | ○ | ○ | ○ | ○ | ○ | ○ | ○ | ○ | ○ | ○ | ○ | ○ | ○ | ○ | ○ | ○ | ○ | ○ | ○ | ○ | ○ | ○ | ○ | ○ | ○ | ○ | ● |

●: The *E. coli* strain used to isolate the phage. Y: The *E. coli* strain could be lysed by the phage. ○: The *E. coli* strain couldn’t be lysed by the phage.

**Table S2. The primers used in this study.**

| **Name** | **Sequence** |
| --- | --- |
| pAmpC01 | CACTCAAACTCAACCATACG GTTTTAGAGCTAGAAATAGCAAG |
| pAmpC02 | ACTAGTATTATACCTAGGACTGAGCTAGCTGTCAAGGATCC |
| pUAmpC1 | CGAGTCGGTGCTTTTTTTGAATTCAGCGGCCAATATCATTGTAAAAGACG |
| pDAmpC1 | AATTCGGACCCGATGGAATTAGGGTCTGGTTTCCATACAA |
| pUAmpC2 | TTGTATGGAAACCAGACCCTAATTCCATCGGGTCCGAATT |
| pDAmpC2 | TAGATCTAAGCTTCTGCAGGTCGACTCCTGTTTCACTTCGTCAGAAATGG |
| AmpCF | GGATCCCGCGATGCACGATCTGAAAAT |
| AmpCR | GAATTCTTACTGTAGAGCGTTGAGAAT |
| Dpo41F | CGCGGATCCATGCAGGTATCACTAG |
| Dpo41R | CCGCTCGAGTTAAATCAAGCCATAAA |
| *E.coli* 16SF | TGCTGCTTTGCTGACGAGTGG |
| *E.coli* 16SR | GGCACATCCGATGGCAAGAGG |
| OmpA-RTF | CCAATCACTGACGACCTGGACATC |
| OmpA-RTR | CTCAACACCGCCAGCGAAGAC |
| OmpAF | CGGAATTCATGAAAAAGACAGCTATCG |
| OmpAR | TCCCCGGGTTAAGCCTGCGGCT |

Primers for PCR were designed using Primer Premier 5 (v5.0). The GC content was about 40-60%, and the difference in Tm values between the upstream and downstream primers was controlled within 10 °C.

**Table S3. Open Reading Frame of phage receptor binding protein.**

| Fragment size | ORF | Full-length | Molecular Mass （KD） | Product |
| --- | --- | --- | --- | --- |
| 1066 | ep3_0031 | 2049 | 74.21 | Phage major capsid protein |
| 267 | ep3_0041 | 2049 | 71.82 | Putative tail fiber protein |

**Table S4. Putative receptors for Dpo41 on *E. coli*.**

| Protein IDs | Peptide counts (unique) | Protein name | Molecular Mass （Da） |
| --- | --- | --- | --- |
| sp\|P69776\|LPP_ECOLI | 2 | Major outer membrane lipoprotein Lpp | 8323 |
| sp\|P0A910\|OMPA_ECOLI | 3 | Outer membrane protein A | 37201 |
| sp\|P0A6P9\|ENO_ECOLI | 3 | Enolase | 45655 |
